# Postoperative urinary retention: risk factors, bladder filling rate and time to catheterization: an observational study as part of a randomized controlled trial

**DOI:** 10.1186/s13741-020-00167-z

**Published:** 2021-01-04

**Authors:** Tammo A. Brouwer, E. N. van Roon, P. F. W. M. Rosier, C. J. Kalkman, N. Veeger

**Affiliations:** 1 Department of Anesthesiology, Medical Center Leeuwarden, Henri Dunantweg 2, PO Box 888, 8901 BR Leeuwarden, The Netherlands; 2 Department of Pharmacotherapy, Epidemiology & Economics, University of Groningen, Groningen, The Netherlands; 3 Department of Clinical Pharmacy & Pharmacology, Medical Center Leeuwarden, Henri Dunantweg 2, PO Box 888, 8901 BR Leeuwarden, The Netherlands; 4 Department of Urology, University Medical Center Utrecht, Utrecht, The Netherlands; 5 Division of Anesthesiology, Intensive Care and Emergency Medicine, University Medical Center Utrecht, Utrecht, The Netherlands; 6 Department of Epidemiology, Medical Center Leeuwarden, Leeuwarden, The Netherlands; 7 Department of Epidemiology, University Medical Center Groningen, Groningen, The Netherlands

**Keywords:** Anesthesia, Catheterization, Maximum Bladder Capacity, Postoperative, Risk Factors, Surgery, Urinary Retention

## Abstract

**Background:**

Knowledge of risk factors for postoperative urinary retention may guide appropriate and timely urinary catheterization. We aimed to determine independent risk factors for postoperative urinary catheterization in general surgical patients. In addition, we calculated bladder filling rate and assessed the time to spontaneous voiding or catheterization. We used the patients previously determined individual maximum bladder capacity as threshold for urinary catheterization.

**Methods:**

Risk factors for urinary catheterization were prospectively determined in 936 general surgical patients. Patients were at least 18 years of age and operated under general or spinal anesthesia without the need for an indwelling urinary catheter. Patients measured their maximum bladder capacity preoperatively at home, by voiding in a calibrated bowl after a strong urge that could no longer be ignored. Postoperatively, bladder volumes were assessed hourly with ultrasound. When patients reached their maximum bladder capacity and were unable to void, they were catheterized by the nursing staff. Bladder filling rate and time to catheterization were determined.

**Results:**

Spinal anesthesia was the main independent *modifiable* risk factor for urinary catheterization (hyperbaric bupivacaine, relative risk 8.1, articaine RR 3.1). *Unmodifiable* risk factors were a maximum bladder capacity < 500 mL (RR 6.7), duration of surgery ≥ 60 min (RR 5.5), first scanned bladder volume at the Post Anesthesia Care Unit ≥250mL (RR 2.1), and age ≥ 60 years (RR 2.0). Urine production varied from 100 to 200 mL/h. Catheterization or spontaneous voiding took place approximately 4 h postoperatively.

**Conclusion:**

Spinal anesthesia, longer surgery time, and older age are the main risk factors for urinary retention catheterization. Awareness of these risk factors, regularly bladder volume scanning (at least every 3 h) and using the individual maximum bladder capacity as volume threshold for urinary catheterization may avoid unnecessary urinary catheterization and will prevent bladder overdistention with the attendant risk of lower urinary tract injury.

**Trial registration:**

**Dutch Central Committee for Human Studies registered trial database**: NL 21058.099.07.

**Current Controlled Trials database**: Preventing Bladder Catheterization after an Operation under General or Spinal Anesthesia by Using the Patient’s Own Maximum Bladder Capacity as a Limit for Maximum Bladder Volume. ISRCTN97786497. Registered 18 July 2011 -Retrospectively registered. The original study started 19 May 2008, and ended 30 April 2009, when the last patient was included.

## Introduction

Post-operative urinary retention (POUR) followed by urinary catheterization is a well-known and frequent complication after surgery under general or spinal anesthesia (Brouwer et al., 2015; Baldini et al., 2009; Darrah et al., 2009; Choi & Awad, 2013). Since the introduction of routine bladder ultrasounds, the definition of ‘POUR necessitating urinary catheterization’ has gradually changed, in that it now relates more to a volume limit (=scanned bladder volume in milliliters) rather than a time limit (=patient must have voided within a certain time period). Reported bladder volume limits in the literature vary from 400 to 600 mL (Wyndaele & De Wachter, 2002; Pavlin et al., 1999). We have previously demonstrated a large interindividual variation in maximum bladder volume, independent of age, gender, and body mass index (BMI) (Brouwer et al., 1999). The beneficial effect of assessing and evaluating the individual maximum bladder capacity (MBC) as a volume/capacity limit for POUR to prevent unnecessary urinary catheterization, was established in a large-scale randomized controlled trial (RCT) (risk reduction 0.73, 95%CI 0.55 to 0.96; *p* = 0.025) (Brouwer et al., 2015).

Even though urinary catheterization is the go-to solution to prevent bladder overdistention, it is an embarrassing procedure that most patients would like to avoid. It can cause urethral trauma, discomfort and urinary tract infection. On the other hand, bladder overdistention can cause temporary or even permanent damage to the lower urinary tract (LUT) (Choi & Awad, 2013; Brouwer et al., 1999; Mason et al., 2016; Nevo et al., 2019). LUT dysfunction can vary from mild (frequent voiding ) to moderate (recurrent urinary tract infections) and can lead to severe adverse events (permanent bladder damage ending in life-long self-catheterization) (Dreyer et al., 2011; Umer et al., 2015; Wu et al., 2012). It is unknown how many patients are affected annually by complications of urinary catheterization or long-term bladder overdistention (> 3 h). Moreover, it is a clinical reality that POUR is handled by the nursing staff, out of sight from the anesthesiologists and surgeons. These facts could explain the lack of urgency and why preventing urinary catheterization and bladder overdistention is not highly ranked on the priority lists of surgeons and anesthesiologists. Executive prevention and management of POUR seems to vary between hospitals and/or countries. Thus, anesthesiologists might feel obliged to take responsibility, whereas POUR may be considered a surgical complication as well. Currently, The American Society of Anesthesiologists (ASA) and the Dutch Society of Anesthesiologists (NVA) have no practice guidelines for the management of POUR.

### Primary aim of the study

To identify risk factors for urinary catheterization in a controlled setting. To this end, we used the data from a previous RCT and considered the individual MBC (rather than a fixed bladder volume limit) as the threshold for urinary catheterization (Brouwer et al., 2015). The strength of the risk factors may vary based on how the need for catheterization is defined. In addition to the identification of risk factors, we calculated the bladder filling rate and analyzed the time to spontaneous voiding or catheterization. The results of these analyses may help health care providers in their decision-making and, as such, prevent unnecessary urinary catheterizations and potential adverse effects on the LUT.

## Methods

### Type of study

This is an observational study analyzing risk factors for urinary catheterization as part of an RCT (Brouwer et al., 2015).

### Participating patients

All patients enrolled in the RCT provided written informed consent, including permission to use data for additional analysis. Included patients were at least 18 years of age and scheduled to undergo a surgical intervention under general or spinal anesthesia. Perioperatively, there was no anticipated need for an indwelling urinary catheter. Patients were informed and asked to participate during their visit at the pre-assessment anesthesia clinic (PAC). After approval and informed consent, patients were requested to go to the restroom to assess the residual bladder volume by ultrasound. At home, the MBC was measured by postponing voiding as long as possible. When a strong urge occurred that could no longer be ignored, patients were encouraged to void in a calibrated bowl (supplied by the hospital) to measure their maximum voided volume; this procedure was repeated three times at different moments during the week. The individual MBC was calculated as the largest voided volume at home minus the residual volume measured at the PAC. All data were recorded in the database.

Postoperatively, the bladder of each included patient was scanned every hour until the MBC was reached, at which point the patient was asked to void. If spontaneous voiding was not possible, urinary catheterization was performed by the nursing staff. A research assistant performed the bladder scans using ultrasound (The BladderScan BVI 9400, Verathon, Bothell, WA, USA). The original aim was to evaluate the effectiveness of using the individual MBC rather than a fixed bladder volume limit of 500 mL as an indicator of bladder overdistention, to prevent unnecessary urinary catheterization.

### Outcome

The pre-planned secondary outcome consisted of analyzing risk factors for urinary catheterization, based on the data from the RCT (Brouwer et al., 2015). Only the data of the MBC group was used for analysis, as they were considered new, implementing a revised definition for POUR to evaluate the need for urinary catheterization. The MBC group consisted of 893 patients who were analyzed in the original RCT for IPSS/QoL (international Prostate Symptoms Score/Quality of Life score) (Fig. . 1), and 43 patients with missing data who were still eligible for risk factor analysis (total 936 patients). Pre- and perioperative patient and procedural characteristics, prospectively collected in the original RCT, were considered as potential risk factors/indicators for the need for urinary catheterization (Choi & Awad, 2013; Mason et al., 2016; Umer et al., 2015). Potential risk factors were divided in *unmodifiable*, not influenceable by anesthesiologists and surgeons, and *modifiable*, i.e., under direct control of anesthesiologists (Table 1); the relative risk (RR) was determined for each risk factor. Duration of surgery is not under direct control of anesthesiologists and is therefore considered *not modifiable*. For developing possible prediction models, the rate of bladder filling (mL/h) was calculated and time to catheterization or spontaneous voiding was assessed.

**Fig. 1:**
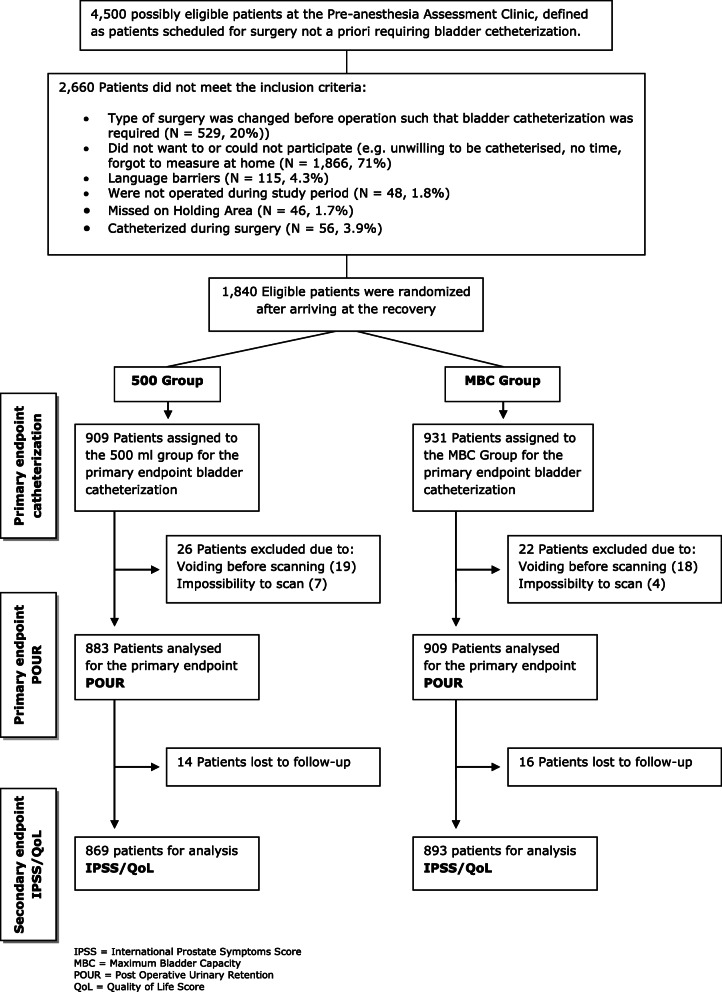
Flow Diagram of the Patients through the Phases of the Randomized Trial

**Table 1:** Unmodifiable and modifiable risk factors

Unmodifiable	Modifiable
Preoperative	
Demographic variables, such as gender and age, BMI, maximum bladder capacity, co-morbidity (hypertension and diabetes) Drugs used, such as beta blockers, benzodiazepines and anti-depressive/anti-psychotic drugs.	Pre-medication with benzodiazepines and/or NSAID’s, Bladder volume/residual volume before start of surgery, time since last voiding.
Perioperative	
Type of surgery, divided in head-neck, thorax/back/breast, lower abdominal or lower extremities, duration of surgery.	Type of anesthesia; general or spinal (divided in short-acting articaine and long-acting bupivacaine), cardiovascular drugs such as atropine, ephedrine, and/or phenylephrine, opioids.
Postoperative	
	Bladder volume after arriving at PACU, total sum of opioids needed**,** drugs given such as cardiovascular therapeutics, opioids, anti-emetics**,** total volume infused or taken.

### Statistical analysis

Categorical data are presented as counts and percentages. Continuous variables are presented as mean with SD or medians with interquartile ranges, depending on normality of the data. For each potential risk factor, differences in the incidence of postoperative urinary catheterization were estimated using a univariate log-binominal regression model. In case of failure to converge, a “modified Poisson” approach was applied with robust error variances to estimate crude relative risks and confidence intervals. After univariate analysis of all potential risk factors, those with a *p* value < 0.10 were included in the initial multivariable model. A backward elimination strategy was used to achieve the most suitable model to estimate the adjusted relative risks with the final multivariable model, only including risk factors associated with postoperative urinary catheterization at a level of *p* < 0.05. In this regard, first order interactions were also taken into consideration. A two-tailed *p* value < 0.05 was considered to indicate statistical significance. All analyses were performed using SAS software, version 9.4 (SAS institute, Inc., Cary, NC, USA).

## Results

A total of 936 surgical patients with complete data on maximum bladder volume entered the study. The average preoperative determined MBC was 611 mL (SD ± 209 mL, range 150 to 1400 mL). The incidence of urinary catheterization was 9.1% (85/936) (Table 2).

**Table 2:** Demographic and clinical characteristics of the study patients

MBC group	***N*** = 936
**Patient data**	
Women, no. (%)	493 (53)
Age, mean (SD), y	47.9 (15)
Height, mean (SD), cm	176 (10)
Weight, mean (SD), kg	81.4 (17)
BMI, mean (SD), kg/m^2^	26.3 (5)
**Type of surgery, no. (%)**	
Head/neck	209 (22)
Thoracic/breast	77 (8)
Spine	33 (4)
Abdominal	273 (29)
Extremities	344 (37)
**Study data**	
MBC, mean (SD), ml	611 (209)
Residual volume, mean (SD), mL	33 (53)
Voided before surgery, no. (%)	877 (94)
Time before surgery, mean (SD), min	59 (48)
Volume at holding, mean (SD), mL	52 (81)
General anesthesia, no. (%)	639 (68)
Spinal anesthesia, no. (%)	297 (32)
Articaine, no. (%)	235 (79)
Bupivacaine, no. (%)	62 (21)
Total volume infused, mean (SD), mL	1,492 (647)
Procedure time, mean (SD), min	61 (40)

*BMI* body mass index, *MBC* maximum bladder capacity, *SD* standard deviation

### Univariate risk factors for urinary catheterization

#### *Modifiable* risk factors

**Fig. 2:**
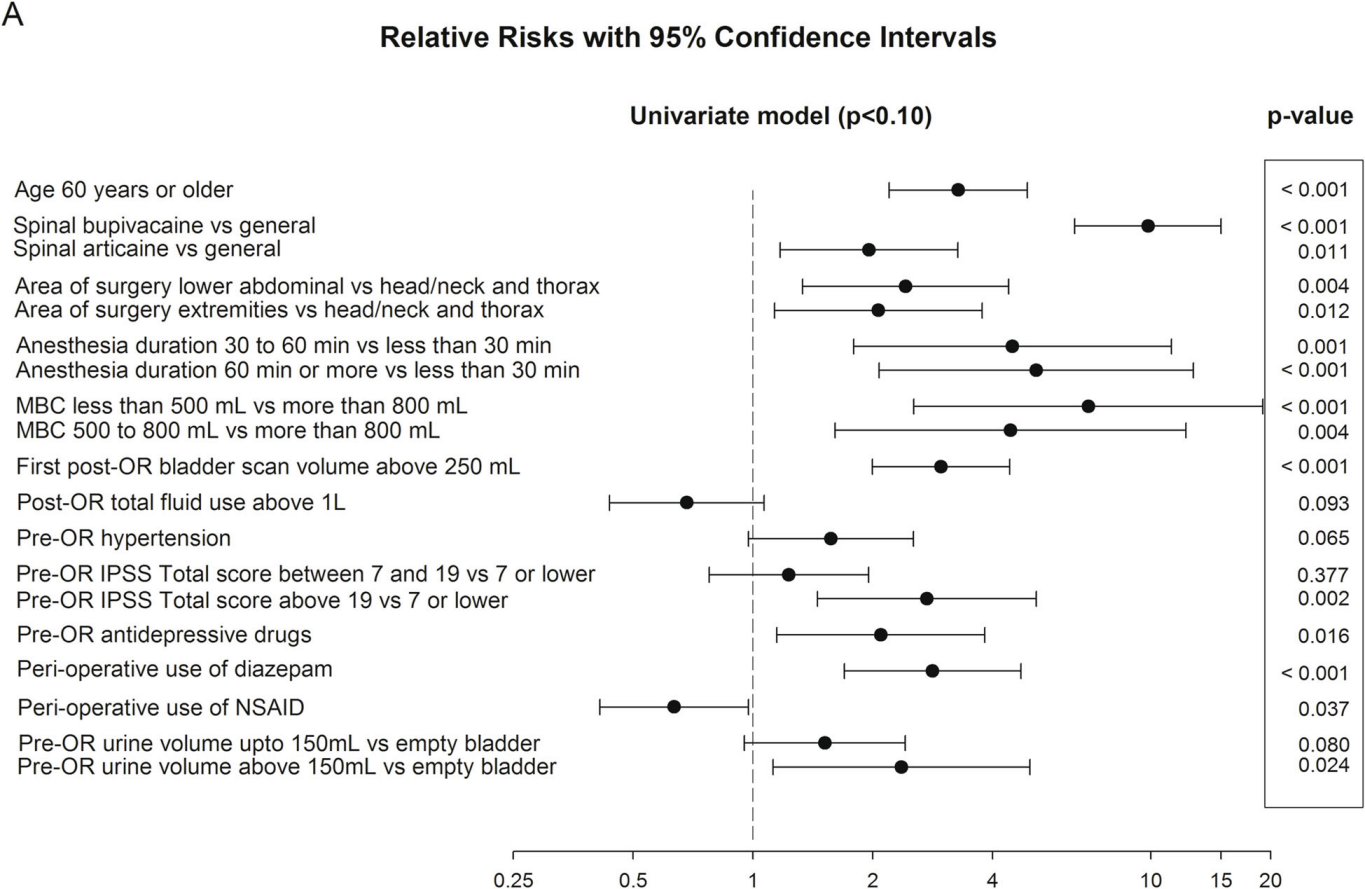
Univariate model. Risk factors, relative risk, *p* value

**Fig. 3:**
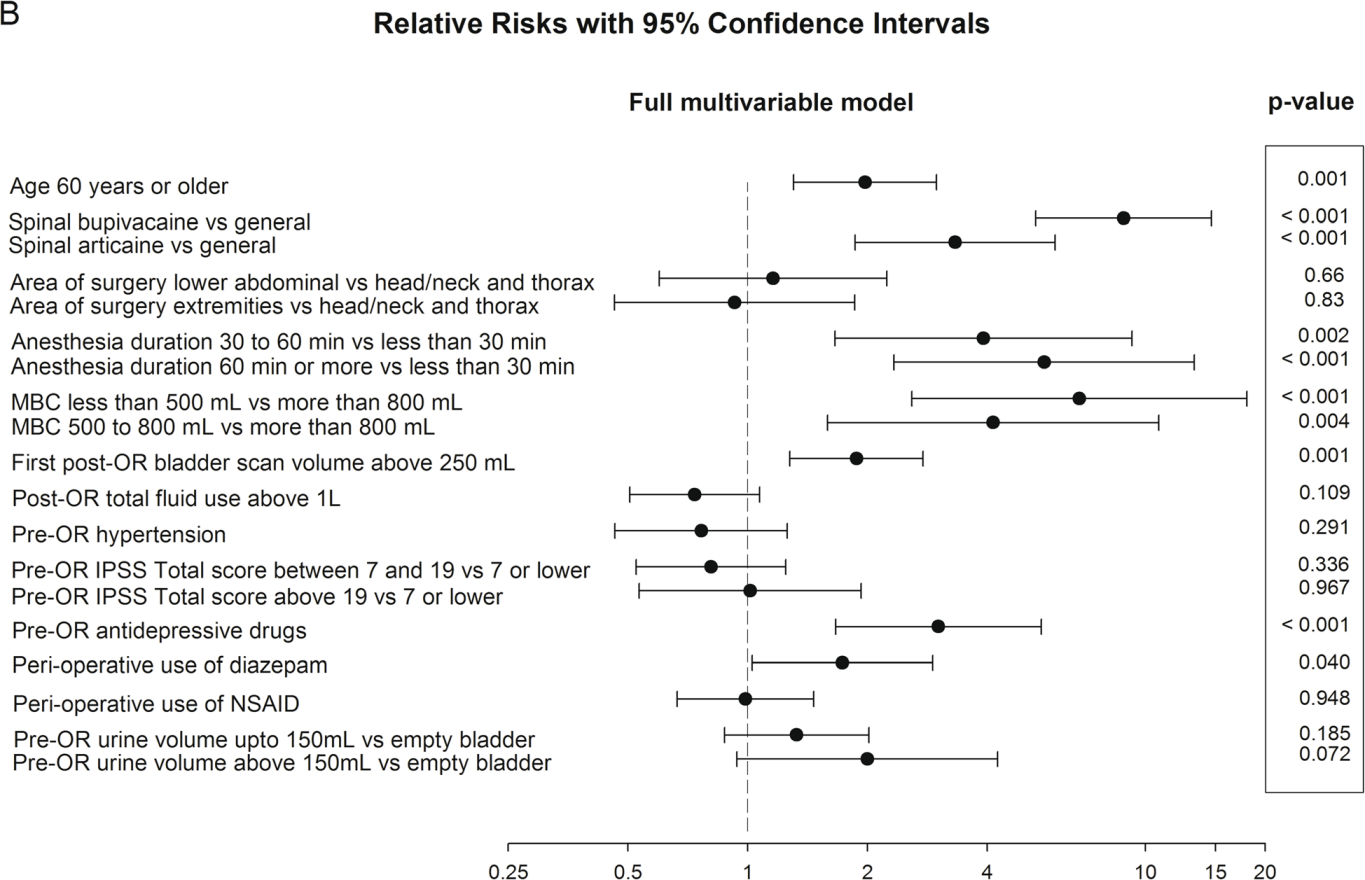
Full multivariable model. Relative risks with 95% confidence intervals. Risk factors, relative risk, *p* value

**Fig. 4:**
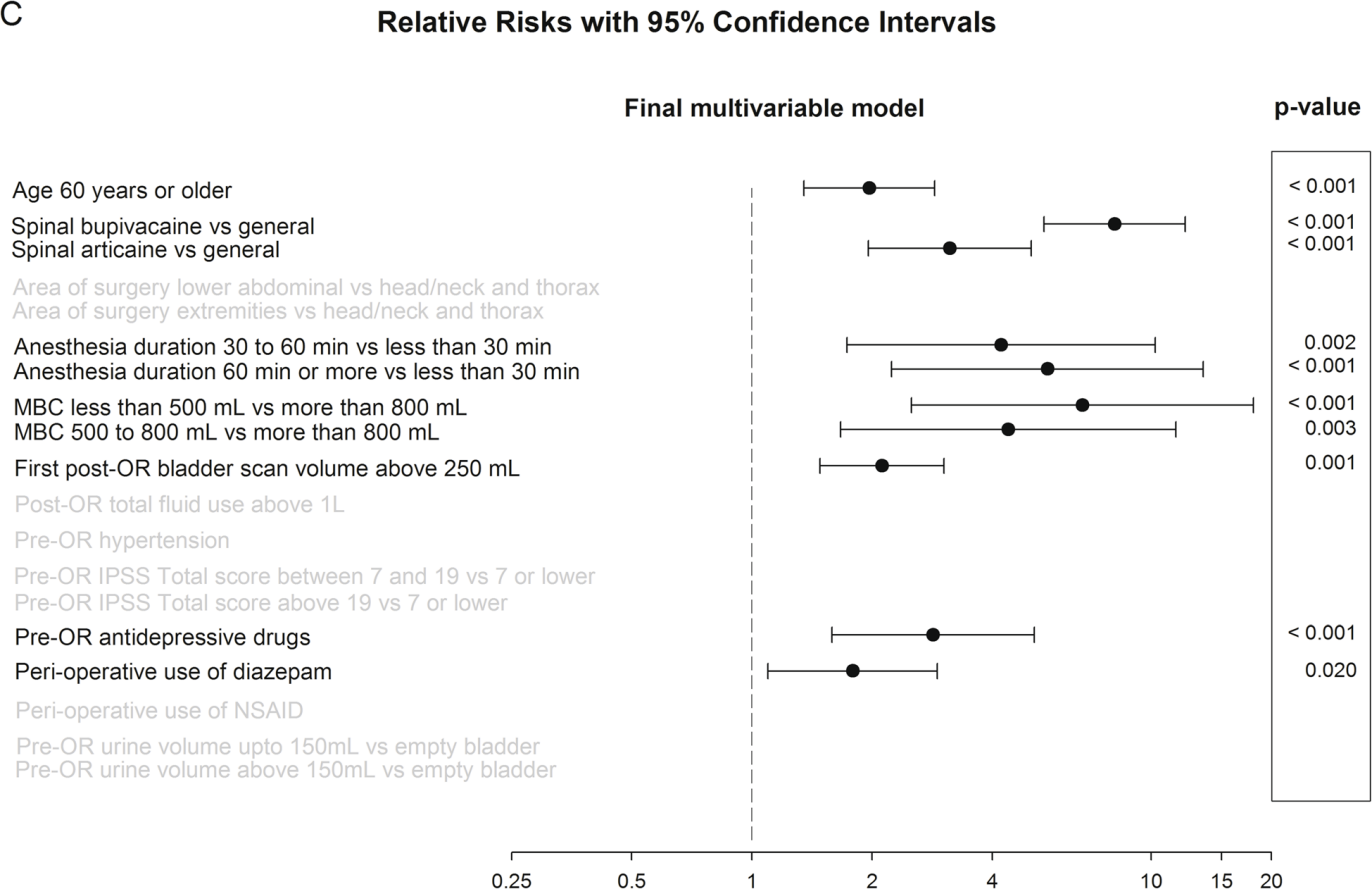
Final multivariable model. Relative risks with 95% confidence intervals. Risk factors, relative risk, *p* value

Figure 2 shows all identified (*un)modifiable* risk factors potentially associated with urinary catheterization (*p* < 0.10). Spinal anesthesia was the strongest *modifiable* risk factor for urinary catheterization. Coupled to spinal anesthesia, and therefore not displayed in Figs. 2, 3 and 4, was the regression of the sensory block. If the sensory block was higher than dermatome T12, voiding was difficult and 69% of these patients had to be catheterized (RR 12.8, 95%CI 8.4 to 18.3; *p* < 0.0001). When the sensory block had regressed below dermatome S3, the incidence was 5.7% (RR 0.8, 95%CI 0.4 to 1.6; *p* = 0.49). A preoperative bladder volume of 150mL or more represented another *modifiable* risk factor (RR_≥ 150 mL_ 2.4, 95%CI 1.6 to 3.5; *p* < 0.02). The total infused volume exceeding 1 L was not a significant risk factor for urinary catheterization (RR 0.7, 95%CI 0.4 to 1.1, *p* = 0.09). Other non-significant risk factors included drugs used perioperatively, e.g., the opioid piritramide (i.v. or s.c.) (RR 1.0, 95%CI 0.7 to 1.6; *p* = 0.91), ephedrine (RR 1.3, 95%CI 0.8 to 2.0, *p* = 0.33) and atropine (RR 1.2, 95%CI 0.7–1.9, *p* = 0.5). For phenylephrine the numbers were too small to analyze.

#### *Unmodifiable* risk factors

A smaller MBC was associated with an increased incidence of urinary catheterization. Of the 300 patients with an MBC < 500 mL, 14% was catheterized as compared to 9% of 398 patients with an MBC between 500 and 800 mL and 2% of 199 patients with an MBC ≥800mL (MBC_< 500 mL_ RR 7.0, 95%CI 2.5 to 19.1; *p* < 0.001). In addition, age ≥ 60 years increased the risk of catheterization (RR 3.3, 95%CI 2.2 to 4.9; *p* < 0.0001), and, when considering the univariate analysis, a higher IPSS was a risk factor as well. In patients with ‘severe’ symptoms (IPSS 20–35 points), the incidence of urinary catheterization was 22% (RR 2.7, 95%CI 1.5 to 5.2, *p* = 0.002).

The strongest *unmodifiable* risk factor ‘related to surgery’ was the duration of surgery (RR_30–60_ 4.5, 95%CI 1.8 to 11.3, RR_> 60_ 5.1, 95%CI 2.1 to 12.8; *p* < 0.001). For the location of surgery, comparing surgeries on head/neck/thoracic (general anesthesia) with those on the abdomen or extremities (general *or* spinal anesthesia), the incidence increased from 4.9 to 11.8 and 10.2%, respectively (RR_abdomen_ 2.4, 95%CI 1.3 to 4.4; *p* < 0.004 and RR _lower extremity_ 2.1, 95%CI 1.1 to 3.7; *p* = 0.012). Another *unmodifiable* risk factor was bladder volume ≥ 250 mL on the first postoperative scan at the PACU (incidence 18.6% compared to 6.3% < 250 mL)(RR 3.0, 95%CI 1.9 to 4.4; *p* < 0.001).

Interestingly, ‘having no urge to void’ when the MBC was reached turned out to be an *unmodifiable* risk factor as well. Of the 84 patients who were catheterized, 60 patients had no urge to void (71%) (RR 4.8, 95%CI 3.1 to 5.9; *p* < 0.001). The influences of gender (RR 0.8, 95%CI 0.5 to 1.2, *p* = 0.31) and existing preoperative hypertension (RR 1.6, 95%CI 1.0 to 2.5, *p* = 0.07) did not reach statistical significance in any of the analyses. Anti-depressant drugs were used by 58 patients (6%) of which 18% was catheterized (RR 2.8; *p* < 0.001), and 61 patients used diazepam (6.5%) of which 23% was catheterized (RR 1.8; *p* = 0.02). For diabetes, the numbers were too small to analyze (26 patients = 3.1%).

### Full multivariable analysis

Figure 3 shows the *full* multivariable analysis for urinary catheterization in the MBC group and includes all potential risk factors with a level of *p* < 0.10 (as determined by the univariate analysis). Using the backward elimination strategy, location of surgery, and ‘severe’ IPSS were not identified as independent risk factors in the multivariable analysis.

### Final multivariable analysis

The *final* multivariable model is displayed in Fig. 4. Spinal anesthesia was the main *modifiable* risk factor with RR values of 8.1 and 3.1 for hyperbaric bupivacaine and articaine, respectively. The *unmodifiable* risk factors MBC (RR 6.7), duration of surgery (RR 5.5), first scan at PACU ≥ 250 mL (RR 2.1), and age ≥ 60 (RR 2.0) were identified as independent risk factors for catheterization.

### Time of voiding or catheterization and rate of bladder filling

Table 3 displays the elapsed time from the start of anesthesia to when patients voided or were catheterized. The rate of bladder filling over this period was estimated by subtracting the preoperative scanned bladder volume from the final scanned bladder volume before spontaneous voiding or catheterization. Both for general and spinal anesthesia, spontaneous voiding occurred after 280 min (4.5 h). The scanned bladder volume amounted to approximately 450 mL with a filling rate of 100 mL/u. Catheterization after general anesthesia was performed significantly later than after spinal anesthesia (352 ± 157 min versus 205 ± 74 min, *p* < 0.001). Spinal anesthesia patients who were catheterized (203 ± 94 mL/h, *p* = 0.005) produced almost twice the amount of urine as those who voided spontaneously (107 ± 63 mL/h).

**Table 3:** Time to catheterization/voiding after general or spinal anesthesia, scanned bladder volumes, and bladder filling rates

	***N***	Mean	Standard deviation	Minimum	Maximum
**General anesthesia**					
**Spontaneous**					
Time (min)	580	282^#^	**±** 117	70	808
Scan volume (mL)	595	412	**±** 206	0	1000
Rate (mL/h)	569	100	**±** 66	0	388
**Catheter**					
Time (min)	26	352^#*^	**±** 157	178	710
Scan volume (mL)	31	602	**±** 216	298	1000
Rate (mL/h)	25	137	**±** 84	32	317
**Spinal anesthesia**					
**Spontaneous**					
Time (min)	238	273^^^	**±** 82	99	712
Scan volume (mL)	238	452	**±** 224	49	999
Rate (mL/h)	234	107^&^	**±** 63	11	379
**Catheter**					
Time (min)	44	205^^*^	**±** 74	99	397
Scan volume (mL)	52	626	**±** 179	330	999
Rate (mL/h)	43	203^&^	**±** 94	94	469

*N*, with missing data, *Time* = time to catheterization or voiding, *Scan volume* = scanned bladder volume before voiding or catheterization, *Rate* = bladder filling rate from start of anesthesia till voiding or catheterization

^#^General anesthesia spontaneous (282 min) versus catheterization (352 min), *p* = 0.032

^^^Spinal anesthesia spontaneous (273 min) versus catheterization (205 min), *p* < 0.001

^*^Spinal anesthesia (205 min) versus general anesthesia (352 min) with catheterization, *p* < 0.001

^&^Spinal anesthesia bladder filling rate, catheterization (203 mL/h) versus spontaneous (107 mL/h), *p* = 0.005

## Discussion

To the best of our knowledge, this is the first study that uses the individual maximum bladder capacity (MBC) to estimate the risk of urinary catheterization after general or spinal anesthesia. In accordance with the literature (Brouwer et al., 2015; Baldini et al., 2009; Mason et al., 2016), the most important *modifiable* risk factor for postoperative urinary catheterization was spinal anesthesia. The risk to be catheterized after hyperbaric bupivacaine and articaine was eight times and three times higher, respectively, as compared to general anesthesia. Available literature comparing general anesthesia with spinal anesthesia and its association with urinary catheterization is limited (Bjerregaard et al., 2015; Fernandez et al., 2014; Niazi & Taha, 2015; Scholten et al., 2018). More specifically, there are no recent studies regarding POUR or urinary catheterization after general anesthesia, let alone comparing their incidence with spinal anesthesia. Most studies about postoperative urinary catheterization are performed in orthopedic patients after spinal anesthesia. During spinal anesthesia, the local anesthetics block the nerves necessary for spontaneous micturition (S2–S4). The spinal block has to regress below dermatome S3 before voluntary control over the external urethral sphincter returns. By then most patients are already able to walk. For bupivacaine, the inability to void may last up to 8 h (Fernandez et al., 2014; Kamphuis et al., 1998). Therefore, if one wishes to reduce the risk for postoperative urinary catheterization (e.g. in day case surgery), it may be justified to change the anesthesia technique. For example, consider using a short-acting local anesthetic for spinal anesthesia, or if possible, use a regional technique (e.g. a femoral or popliteal nerve block), or choose general anesthesia.

Our analysis revealed that an MBC of less than 500 mL was an *unmodifiable* risk factor for urinary catheterization (RR 6.7). Bjerregaard et al. (2015), studying orthopedic patients after fast-track hip or knee surgery, compared a threshold for POUR of 800 versus 500 mL. They found an incidence of 13.4% versus 32.2%. They concluded that a threshold of 800 mL can be set safely, without increasing urological complications (Bjerregaard et al., 2016). Of note, their patient group consisted of ‘older’ patients, with unknown MBC and voiding history. A threshold of 800 mL may lead to complications in patients with lower MBCs (e.g., < 500 mL) or in patients with pre-existing LUT complaints. In general, a strict POUR protocol should be implemented to prevent bladder overdistention. When the MBC is known, the need for urinary catheterization can be precisely determined and this may prevent unnecessary application of the procedure.

Duration of surgery constituted a strong *unmodifiable* risk factor (RR 5.1) in all analyses, consistent with similar studies (Ringdal et al., 2003; Alsaidi et al., 2013; Miller et al., 2013). This could be due to a higher cumulative dose of anesthetic drugs, longer unnoticed bladder filling, or, when using long-acting local anesthetics for spinal anesthetics, an inability to void persisting for more than 8 h. Obtaining shorter surgery times can help to lower the incidence of urinary catheterization.

Not voiding before the start of surgery is considered a *modifiable* risk factor for POUR followed by urinary catheterization. In the univariate analysis, a *pre*operative bladder volume ≥ 150 mL was a significant risk factor, but this significance was not sustained in the final multivariable model. Joelsson-Alm found in her prospective study on bladder distention in orthopedic surgery, that a higher preoperative bladder volume is a risk factor for POUR and urinary catheterization (Joelsson-Alm et al., 2009). She concluded that encouraging patients to void before leaving for operating theatre does not necessarily mean an empty bladder at the start of surgery. Our results confirmed that observation. Patients were at risk for large bladder volumes postoperatively, if they already had a considerable bladder filling at the start of surgery. Indeed, a postoperative bladder volume ≥ 250 mL after the first scan at the PACU was an important *unmodifiable* risk factor (Shadle et al., 2009). Measuring bladder volumes shortly before surgery in the holding area and urging patients to void if needed, can thus contribute to preventing large postoperative bladder volumes (Keita et al., 2005). Interestingly, the absence of urge does not mean an empty bladder; of the patients who felt no urge to void but reached POUR, 71% needed to be catheterized.

In patients ≥ 60 years of age the incidence of urinary catheterization amounted to 18.5%, compared to just 5.7% in subjects < 60 years. Increasing age is a well-known *unmodifiable* risk factor for postoperative urinary catheterization (RR 2.0) (Brouwer et al., 2015; Baldini et al., 2009; Darrah et al., 2009; Choi & Awad, 2013; Kreutziger et al., 2010; Luger et al., 2008; Verhamme et al., 2008). This could be due to higher IPSS scores in older patients. Or possibly the different types of surgery performed in more senior patients (e.g., more surgery on lower abdomen or lower extremities, longer operation times, use of long-acting spinal anesthesia, and the use of ephedrine/atropine). The impact of age ≥ 60 was evident in the univariate analysis but did not reach significance in the final multivariable analysis (Figs. 2 and 4).

The *modifiable risk factor* ‘volume infused and taken orally’ volume exceeding one liter appeared to have a small risk reducing effect, but this was not significant (RR 0.7, *p* < 0.09). Patients had received on average 1.5 L of fluid at the time of voiding or catheterization. In the literature, the amount of fluids infused was considered a *modifiable* risk factor for urinary catheterization (Shadle et al., 2009; Kowalik & Plante, 2016; Keita et al., 2005). More recent studies confirmed that the amount of fluids given or taken perioperatively is not a significant risk factor for urinary catheterization (Brouwer et al., 2015; Scholten et al., 2018; Miller et al., 2013).

Possible *modifiable* risk factors for catheterization are drugs given perioperatively (Baldini et al., 2009; Darrah et al., 2009; Verhamme et al., 2008). Opioids can have a dual effect on voiding; direct—by partially inhibiting the parasympathetic nerves that innervate the bladder, and indirect—by decreasing the awareness of a full bladder and the sensation of urge. Our results could not confirm that piritramide had an effect on the incidence of urinary catheterization (RR 1.0, *p* = 0.91). We did not register pain scores as they were titrated below a VAS of four (visual analog scale), following protocol. Cardiovascular drugs may also affect bladder function through interactions with the sympathetic and parasympathetic nerve system. For atropine and ephedrine this effect was not significant. However, preoperative use of anti-depressant drugs or diazepam did have a significant effect on POUR, although the numbers were relatively small. These patients may need to be monitored more closely.

To estimate the rate of bladder filling after surgery, the time from the start of anesthesia to catheterization or spontaneous voiding was calculated (Table 3). A similar approach has been applied previously by Kreutziger et al. (Kreutziger et al., 2010). They studied time to voiding in 86 patients after spinal anesthesia. On average, catheterization was performed after 200 min and voiding occurred after 270 min (3.5 h), comparable with our findings for spinal anesthesia. In our study, catheterization after general anesthesia was performed later, after almost 6 h. This difference in time to catheterization between spinal and general anesthesia can possibly be explained by a difference in bladder filling rate. In patients who were catheterized the bladder filling rate following spinal anesthesia was almost 70 mL/h *higher* than during general anesthesia (203 mL/h versus 137 mL/h). Bladder filling rate does not only depend on the anesthesia technique, but likely on factors such as age, amount of fluids infused, antidiuretic hormone production, blood pressure, and is probably not linear. More targeted studies are necessary to confirm or refute our results. Yet, considering urine production, and time to catheterization, it is highly recommendable to scan the bladder within 3 h (180 min) after the end of surgery to prevent bladder overdistention. When assessing bladder filling state within this timeframe, some patients may have already reached their MBC, with bladder volumes varying from 300 to 540 mL. This is still a safe margin for urinary catheterization if the MBC is unknown; the procedure may be performed a bit prematurely, but, more importantly, not too late. A full bladder extended beyond its maximum capacity for 2 to 3 h can damage the detrusor muscle and should therefore be avoided at all times (Gosling et al., 2000).

In conclusion, in the present study, we identified important independent risk factors for urinary catheterization. We used the individual maximum bladder capacity as the cut-off bladder volume limit for catheterization. Spinal anesthesia was the most important *modifiable* risk factor, whereas a MBC < 500 mL, duration of surgery ≥ 60 min, the first scan at the PACU ≥ 250 mL, and age ≥ 60 years constituted significant *unmodifiable* risks. Awareness of these risk factors for POUR can help anesthesiologists, surgeons, and the nursing staff to decide when catheterization is necessary (Dal Mago et al., 2010). On average, voiding or catheterization took place 4 h after surgery and the bladder filling rate varied between 100 and 200 mL/h, depending on the anesthesia technique. To prevent injury to the lower urinary tract, a simple algorithm can be considered: (1) preoperatively, at the pre-assessment clinic, ask patients at risk to measure their MBC at home; (2) use this individual MBC as a bladder volume limit throughout the postoperative phase; (3) preoperatively, at the holding area, check if patients have voided before surgery and consider measuring residual bladder volume with bladder ultrasound; (4) if possible, prevent long-acting local anesthetics for spinal anesthesia; (5) postoperatively, perform bladder ultrasound at regular intervals and estimate when the MBC will be reached, knowing bladder filling rate; and (6) implement a POUR protocol at the PACU and the surgical wards, until spontaneous voiding or urinary catheterization is deemed necessary.

Anesthesiologists and surgeons together, should raise awareness among the nursing staff how to recognize POUR and when to perform urinary catheterization when necessary.

## Data Availability

The datasets used and analyzed during the current study are available from the corresponding author on reasonable request (t.brouwer@mcl.nl).

## Abbreviations

RCT: Randomized controlled trial
POUR: Postoperative urinary retention
LUT: Lower urinary tract
MBC: Maximum bladder capacity
PAC: Pre-assessment anesthesia clinic
PACU: Post anesthesia care unit
IPSS: International Prostate Symptoms Score
QoL: Quality of life
